# Umbilical Artery Doppler Indices in Hypertensive Disorders of Pregnancy: Impact on Fetal Outcomes

**DOI:** 10.7759/cureus.50876

**Published:** 2023-12-20

**Authors:** Kalyani S Mahajan, Deepika Dewani, Sakshi Duragkar

**Affiliations:** 1 Obstetrics and Gynecology, Symbiosis Medical College for Women, Symbiosis International University, Pune, IND; 2 Obstetrics and Gynecology, Jawaharlal Nehru Medical College, Datta Meghe Institute of Higher Education and Research, Wardha, IND

**Keywords:** pulsatility index (pi), resistance index (ri), systolic/diastolic (s/d) ratio, adverse fetal outcomes, umbilical artery doppler, hypertensive disorders of pregnancy (hdp)

## Abstract

Background

Hypertensive disorders of pregnancy (HDP) are associated with increased maternal and fetal risks. Doppler ultrasound indices of the umbilical artery have shown promise in predicting adverse fetal outcomes in HDP patients. This observational study investigated the correlation between abnormal umbilical artery Doppler indices and adverse fetal outcomes in HDP patients.

Methodology

Over a two-year period from 2020 to 2022, in Acharya Vinoba Bhave Rural Hospital, central India, we enrolled 138 pregnant women with HDP beyond 28 weeks of gestation and singleton pregnancies. Comprehensive clinical assessments, laboratory investigations, and Doppler studies of the umbilical artery were performed. Doppler indices assessed included the systolic/diastolic (S/D) ratio, resistance index (RI), and pulsatility index (PI). Adverse fetal outcomes were defined based on birth weight and neonatal intensive care unit admissions. Chi-square or Fisher’s exact test was used for analyzing the relationship between qualitative data, while an independent-sample t-test was employed for quantitative data.

Results

Abnormal umbilical artery Doppler indices, including an elevated S/D ratio, RI, and PI, demonstrated a positive correlation with adverse fetal outcomes in HDP patients. These findings highlight the significance of umbilical artery Doppler indices as reliable indicators for anticipating adverse fetal outcomes in HDP patients.

Conclusions

Abnormal Doppler indices in the umbilical artery, including an elevated S/D ratio, RI, and PI, appear to be valuable predictors for adverse fetal outcomes in patients with HDP. Monitoring these indices can aid in risk stratification and improve the management of pregnancies complicated by HDP.

## Introduction

Hypertensive disorders of pregnancy (HDP) pose a significant and complex challenge to maternal and fetal well-being, representing a leading cause of maternal and perinatal morbidity and mortality worldwide [1]. These disorders, which encompass conditions such as gestational hypertension, preeclampsia, and eclampsia, are characterized by elevated blood pressure during pregnancy and often lead to a cascade of adverse maternal and fetal consequences. The intricate interplay of maternal health and fetal outcomes in the context of HDP necessitates comprehensive assessment tools to guide clinical management [2].

Among the diagnostic modalities used to evaluate the impact of HDP on fetal well-being, Doppler ultrasound studies of the umbilical and middle cerebral arteries have gained prominence. These studies enable the non-invasive assessment of blood flow patterns in the umbilical artery, which supplies oxygenated blood to the fetus, and the middle cerebral artery, which reflects cerebral perfusion in the fetal brain. Aberrations in these Doppler indices are indicative of compromised fetal hemodynamics and have been associated with adverse fetal outcomes [3].

This observational study aims to investigate the role of umbilical artery Doppler indices as prognostic markers for fetal outcomes in patients with HDP. By exploring the relationships between abnormal Doppler findings and key fetal outcomes, including low birth weight (LBW) infants and the requirement for neonatal intensive care unit (NICU) admission, this research seeks to shed light on the predictive value of these Doppler assessments in HDP [4].

Understanding the impact of umbilical artery Doppler indices on fetal outcomes in the context of HDP is crucial for obstetric care. These indices provide valuable insights into fetal well-being and can aid clinicians in identifying pregnancies at higher risk for adverse outcomes. Consequently, this knowledge may inform more tailored monitoring and interventions, ultimately leading to improved maternal and fetal outcomes in the challenging landscape of HDP.

## Materials and methods

Study design, population, and duration

This study adhered to an observational design and was conducted at the Department of Obstetrics and Gynecology, Acharya Vinoba Bhave Rural Hospital, Sawangi (Meghe), Wardha, Maharashtra, India. The study spanned a duration of two years (2020-2022). It focused on pregnant women experiencing HDP, specifically those carrying singleton pregnancies with a gestational age surpassing 28 weeks and presenting abnormal Doppler studies, following the defined inclusion and exclusion criteria.

Sample size calculation

The determination of the sample size for this study was predicated on the anticipated occurrence rate of HDP within the geographical scope of the research site, which was estimated to be 10%. Employing a 5% type I error rate (with a critical value of Z_(1-∝/2) equal to 1.96), the sample size was calculated using Yamane’s formula: N = (Z_(1-∝/2)^2 × p × (1 - p))/d^2, where p represents the incidence of HDP at 10% (0.1) [4] and D is the desired margin of error at 5% (0.05). Subsequently, the calculated sample size was determined to be 138 individuals.

Data collection

The data collection process involved securing written informed consent from participants in the local language (Marathi), documented through a predesigned and pretested structured proforma. This proforma encompassed comprehensive information concerning medical history, clinical examinations, and ultrasonography. Patients eligible for the study were selected based on specific criteria. Inclusion criteria comprised hypertensive pregnant women beyond 28 weeks of gestation, those with singleton pregnancies, and those diagnosed with HDP exhibiting abnormal Doppler parameter changes. Exclusion criteria included pregnant women with various medical complications such as renal disorders, liver disorders, diabetes mellitus, heart disease, autoimmune disorders, chronic illnesses, multiple pregnancies, assisted conception, and congenital anomalies.

Pregnant women were recruited to meet the eligibility criteria following valid informed consent. The study proforma collected demographic information while detailed clinical and obstetrical examinations were conducted. Laboratory investigations adhered to protocols for HDP. The Department of Radiodiagnosis performed an obstetric ultrasound and color Doppler examinations using the ALOKA HITACHI ARIETTA 65 - G3003853 ultrasound unit. Evaluating Doppler indices for the umbilical artery involved assessing parameters such as the systolic/diastolic (S/D) ratio, resistance index (RI), and pulsatility index (PI).

The examination of the umbilical artery occurred at mid-cord or placental insertion, with abnormal values determined as S/D ratio >3, RI and PI exceeding the 95th percentile, or the presence of absent or reverse end-diastolic flow. Follow-up Doppler studies were conducted as needed to monitor fetal well-being. The analysis utilized the results of the last Doppler ultrasound within one week of delivery. Management decisions were based on clinical status and Doppler ultrasound reports, including the termination of pregnancy when necessary. Patients were followed up until delivery and fetal outcomes were studied. Adverse fetal outcomes were categorized based on variables such as birth weight and NICU admissions, with all neonates weighing below 2.5 kg or requiring NICU admission classified as having adverse fetal outcomes.

Ethical clearance and informed consent

Ethical clearance for the study was obtained from the Ethical Review Committee of Datta Meghe University of Medical Sciences, Wardha (approval number: IEC/2020/9059). Informed written consent was obtained from all eligible participants after explaining the methodology and relevance of the study in detail.

Data analysis

Data were analyzed using SPSS version 24.0 (IBM Corp., Armonk, NY, USA). Qualitative data were expressed as percentages, and quantitative data were presented as mean ± SD and range. The chi-square or Fisher’s exact test was used for analyzing the relationship between qualitative data, while an independent-sample t-test was employed for quantitative data. Sensitivity, specificity, positive predictive value (PPV), negative predictive value (NPV), and diagnostic accuracy were calculated. A p-value less than 0.05 was considered statistically significant.

## Results

Table 1 summarizes the demographic characteristics of patients in a study on HDP with Doppler changes. Among the 138 patients, the age distribution ranged from ≤19 to over 35 years. Body mass index (BMI) classifications included underweight (2.2%), normal (83.3%), and overweight (14.5%), with no cases of obesity. Hypertensive disorders comprised gestational hypertension (41.3%), preeclampsia (53.6%), and eclampsia (5.1%). Primigravida accounted for 67.4%, and multigravida 32.6%. Delivery gestational age varied, with 15.9% at 28-32.6 weeks, 51.5% at 33-36.6 weeks, and 32.6% at 37-40 weeks.

**Table 1:** Distribution of patients based on the demographic variable in HDP with Doppler changes. HDP: hypersensitive disorders of pregnancy; BMI: body mass index

Demographic characteristics	Number of patients	Percentage
Age group		
≤19 years	4	2.9
20–24 years	60	43.5
25–29 years	47	34.1
30–35 years	24	17.4
35 years	3	2.2
Total	138	100.0
BMI		
<18.5 (underweight)	3	2.2
18.5–24.9 (normal)	115	83.3
25–29.9 (overweight)	20	14.5
≥30 (obese)	0	0.0
Total	138	100.0
Hypertensive disorder of pregnancy		
Gestational hypertension	57	41.3
Chronic hypertension	0	0.0
Preeclampsia	74	53.6
Eclampsia	7	5.1
Chronic hypertension with superimposed preeclampsia	0	0.0
Total	138	100.0
Parity		
Primigravida	93	67.4
Multigravida	45	32.6
Total	138	100.0
Gestational age at delivery		
28–32.6 weeks	22	15.9
33–36.6 weeks	71	51.5
37–40 weeks	45	32.6
Total	138	100.0

Table 2 presents the distribution of umbilical artery Doppler indices in HDP. For the S/D ratio, 76.08% of patients had a normal index, while 23.92% showed abnormal values. Regarding the PI, 78.3% exhibited a normal index, with 21.7% displaying abnormal values. Regarding the RI, 65.95% had a normal index, while 34.05% had abnormal values, offering insights into the prevalence of abnormal Doppler indices in the umbilical artery among patients with HDP.

**Table 2:** Distribution of umbilical artery Doppler indices in HDP. S/D ratio: systolic/diastolic ratio; PI: pulsatility index; RI: resistance index; HDP: hypertensive disorders of pregnancy

Distribution		Number of patients	Percentage
Umbilical artery indices			
S/D ratio	Normal	105	76.08
	Abnormal	33	23.92
PI	Normal	108	78.3
	Abnormal	30	21.7
RI	Normal	91	65.95
	Abnormal	47	34.05

Table 3 summarizes delivery and fetal outcomes in the study on HDP with Doppler changes. Delivery modes included 41.3% normal vaginal delivery, 5.8% instrumental delivery, and 52.9% lower segment cesarean section. For birth weight, 7.9% were extremely low, 12.4% very low, 42.8% low (LBW), and 36.9% normal (>2.5 kg). NICU admissions were needed for 57.25% of cases, while 42.75% did not require admission, which overviews delivery and fetal outcomes among HDP patients with Doppler changes.

**Table 3:** Distribution of patients based on delivery and fetal outcome details in HDP with Doppler changes. NVD: normal vaginal delivery; LSCS: lower segment cesarean section; ELBW: extremely low birth weight (<1 kg); VLBW: very low birth weight (1-1.5 kg); LBW: low birth weight (1.5-2.5 kg); NICU: neonatal intensive care unit; HDP: hypertensive disorders of pregnancy

Characteristics	Number of cases	Percentage
Mode of delivery		
NVD	57	41.3
Instrumental delivery	8	5.8
LSCS	73	52.9
Total	138	100.0
Birth weight		
ELBW (<1 kg)	11	7.9
VLBW (1–1.5 kg)	17	12.4
LBW (1.5–2.5 kg)	59	42.8
Normal (>2.5 kg)	51	36.9
Total	138	100.0
NICU admissions		
Yes	79	57.25
No	59	42.75
Total	138	100.0

Table 4 demonstrates the correlation between abnormal umbilical artery Doppler indices and fetal outcomes in HDP. The parameters studied include the S/D ratio, RI, and PI. Adverse outcomes were observed in cases with abnormal values, while good outcomes were associated with normal values. Statistical analysis using the chi-square test revealed significant associations for all three parameters, with p-values of 0.01 indicating statistical significance. Table 4 highlights the link between abnormal umbilical artery Doppler indices and adverse fetal outcomes in HDP.

**Table 4:** Association between abnormal umbilical artery Doppler indices with adverse fetal outcome in HDP. S/D ratio: systolic/diastolic ratio; RI: resistance index; PI: pulsatility index; X^2^: chi-square test statistic; S: statistically significant; HDP: hypertensive disorders of pregnancy

Umbilical artery parameters		Adverse outcome	Good outcome	X^2^	P-value
S/D ratio	Abnormal	25	8	19.52	0.01, S
	Normal	18	87		
RI	Abnormal	23	24	12.61	0.01, S
	Normal	18	73		
PI	Abnormal	19	11	40.21	0.01, S
	Normal	21	87		

Table 5 highlights the correlation between umbilical artery status and adverse fetal outcomes in HDP. The adverse outcomes, including low APGAR scores, NICU admission, LBW, and mortality, were significantly more prevalent in cases with abnormal umbilical artery findings (N = 46) compared to those with normal status (N = 92). The chi-square test indicated statistical significance (p < 0.01) for all outcomes. It is important to note that one neonate could experience multiple adverse outcomes. The results emphasize the strong association between abnormal umbilical artery status and adverse fetal outcomes in HDP.

**Table 5:** Correlation of umbilical artery with adverse fetal outcomes in HDP. *: One neonate may show multiple outcomes. APGAR: appearance, pulse, grimace, activity, respiration; NICU: neonatal intensive care unit; LBW: low birth weight; HDP: hypertensive disorders of pregnancy

Fetal outcomes	Umbilical artery				P-value
	Abnormal (n = 46)	%	Normal (n = 92)	%	
APGAR	20	43.48	8	8.69	<0.01, S
NICU	26	56.52	12	13.04	<0.01, S
LBW	21	45.65	4	4.35	<0.01, S
Mortality	4	8.69	0	0	<0.01, S

Table 6 assesses the predictive accuracy of abnormal umbilical artery Doppler indices for adverse fetal outcomes in HDP. The parameters analyzed include umbilical artery systolic/diastolic (UA S/D), umbilical artery pulsatility index (UA PI), and umbilical artery resistance index (UA RI). UA S/D showed a sensitivity of 58.13%, specificity of 91.58%, PPV of 75.76%, NPV of 82.86%, and overall diagnostic accuracy (DA) of 81.10%. UA PI had a sensitivity of 51.11%, specificity of 93.55%, PPV of 79.31%, NPV of 79.82%, and DA of 79.71%. UA RI exhibited a sensitivity of 55.56%, specificity of 73.53%, PPV of 42.55%, NPV of 84.42%, and DA of 68.84%. The result summarizes the diagnostic performance of abnormal umbilical artery Doppler indices in predicting adverse fetal outcomes in HDP.

**Table 6:** Accuracy of abnormal UA Doppler indices in anticipating adverse fetal outcome in HDP. UA: umbilical artery; S/D: systolic/diastolic; PI: pulsatility index; RI: resistance index; PPV: positive predictive value; NPV: negative predictive value; DA: diagnostic accuracy; HDP: hypertensive disorders of pregnancy

Artery	Parameter	Sensitivity	Specificity	PPV	NPV	DA
Umbilical artery	UA S/D	58.13	91.58	75.76	82.86	81.10
	UA PI	51.11	93.55	79.31	79.82	79.71
	UA RI	55.56	73.53	42.55	84.42	68.84

## Discussion

The incidence of HDP underscores regional disparities influenced by factors such as race, socioeconomic status, and demographic parameters. In this study, the HDP incidence was 10%, with preeclampsia emerging as the most common subtype. Comparative analyses with other Indian studies, including Panda et al. (2021) [5], Sachdeva et al. (2011) [6], and Vidyadhar et al. (2011) [7], provide context to these findings. The association between maternal age and HDP reveals a notable occurrence in younger women, especially in rural areas where early marriage prevails. The mean age of HDP cases in the study aligns with Gaikwad et al. [8] and Mishra et al. [9].

Socioeconomic factors and rural residence influence HDP prevalence, with lower socioeconomic status linked to a higher incidence, possibly due to limited access to antenatal care. These findings echo those of Lakhute et al. [10]. The study establishes a connection between BMI and HDP, indicating a higher risk with increased BMI. Primigravida women are identified as having a higher risk, consistent with the study by Sibai et al. [11].

Gestational age at delivery is pivotal in HDP, with over half delivering before 37 weeks, aligning with similar findings in other studies. Preterm delivery is associated with severe hypertension, as reported by Buchbinder et al. [12]. The significance of uterine artery Doppler indices is highlighted, noting common abnormalities in S/D ratio, PI, RI, and notches in HDP. Comparisons with studies by Nagar et al. [13], Fleischer [14], Konwar et al. [15], and Parmar et al. [16] underscore variations in abnormal Doppler indices.

The study underscores the importance of umbilical artery Doppler indices in predicting fetal outcomes, discussing abnormalities in S/D ratio, PI, and RI. Comparisons with Nagar et al. [13], Gaikwad et al. [8], Sharma et al. [17], Adekanmi et al. [18], and Parmar et al. [16] highlight variations in mean values and abnormal Doppler ratios. The significance of middle cerebral artery (MCA) Doppler indices in assessing fetal oxygenation status is explored, examining abnormalities in MCA S/D ratio, PI, and RI. Comparisons with Parmar et al. [16], Gaikwad et al. [8], and Sharbaf et al. [19] are made.

The study investigates labor type and mode of delivery in HDP, reporting rates of spontaneous and induced labor and a high percentage of cesarean deliveries. Findings align with Aharwal et al.’s [20] observation that cesarean deliveries significantly increase in women with hypertension and Doppler changes. APGAR scores, birth weight, NICU admissions, and mortality rates are analyzed. Low APGAR scores are linked to uteroplacental insufficiency, low birth weights are prevalent in HDP, and NICU admissions are common due to causes such as low birth weight and prematurity. Fetal mortality is discussed, with comparisons to studies such as Mohan et al. [21] and Konwar et al. [15]. The correlation between abnormal Doppler indices and adverse fetal outcomes is analyzed, reporting sensitivity, specificity, PPV, NPV, and DA for various Doppler indices. Pre-diastolic notching in the uterine artery is identified as an accurate predictor of adverse fetal outcomes in hypertensive pregnant women with abnormal Doppler ultrasound.

Limitations

Strengths of the study include its comprehensive analysis of Doppler indices and their correlation with adverse fetal outcomes. However, limitations are acknowledged, such as its observational nature and the single-center design. Future multi-center, prospective studies replicated in other geographies could further validate these findings and enhance their generalizability.

## Conclusions

Our study underscores the importance of abnormal umbilical artery Doppler indices as reliable predictors for anticipating adverse fetal outcomes in individuals with HDP. Deviations in S/D ratio, RI, and PI in these arteries demonstrated positive associations with adverse fetal outcomes, highlighting their potential clinical significance. These results propose that monitoring umbilical artery Doppler indices can contribute to risk stratification and enhance the management of pregnancies complicated by hypertensive disorders.
